# Real experiences and care needs of frail older patients: a systematic review of qualitative studies

**DOI:** 10.3389/fpubh.2025.1679832

**Published:** 2025-10-01

**Authors:** Shuo Peng, Hongzhi He, Xi Luo, Jingping Kang, Xinyi Wang, Yongqiong Tan

**Affiliations:** ^1^Department of Anesthesiology, West China Hospital of Sichuan University, Chengdu, Sichuan, China; ^2^Department of Anesthesiology, West China Tianfu Hospital, Sichuan University, Chengdu, Sichuan, China; ^3^Operating Room Department, West China Tianfu Hospital, Sichuan University, Chengdu, Sichuan, China

**Keywords:** frailty, older adults, experience, needs, qualitative research, meta-synthesis

## Abstract

**Objective:**

By conducting a meta-synthesis of qualitative research, this study evaluates the real experiences and care needs of frail older patients, providing valuable references and insights for care service providers, policymakers, and medical researchers in developing intervention programs.

**Design:**

PubMed, Web of Science, Scopus, Embase, CINAHL, and PsycINFO were searched from the establishment of the databases to August 25, 2024.

**Setting:**

The real experiences and care needs of frail older patients are gradually becoming the focus of social attention. Qualitative research is of great significance for improving the quality of care services for such patients in the future.

**Outcome measures:**

The quality of the studies included was appraised using the Joanna Briggs Institute Quality Assessment of Qualitative Research criteria. Data extraction was performed using NVivo (v14), and results were synthesized using a meta-synthesis approach.

**Results:**

511 frail older patients were included in 15 studies. Three main themes were identified: the negative impact of frailty on the physical, psychological, and behavioral aspects of older patients; positive attitudes toward the current situation among frail older patients; and the multidimensional care needs of frail older patients.

**Conclusion:**

Frail older adults face multidimensional challenges in physiology, psychology, and behavior, and have care needs in areas such as professional services and social support.

**Systematic review registration:**

https://www.crd.york.ac.uk/PROSPERO/view/CRD420251038626.

## 1 Introduction

Frailty is a complex clinical condition associated with aging and caused by multiple factors. Frailty is defined as the deterioration of bodily functions and age-related loss of reserve capacity, leading to a reduced ability to cope with internal and external stressors (1, 2). In recent years, with the rapid growth of the aging population, the prevalence of frailty among older individuals has increased significantly. A survey study on the time trend of frailty prevalence among middle-aged and older people in China from 2011 to 2020 showed that the standardized prevalence increased from 13.5% in 2011 to 16.3% in 2020 (3). The prevalence rates of frailty and pre-frailty among old inpatients in low- and middle-income countries reached 39.1 and 51.4%, respectively (4). The high prevalence of frailty exacerbates adverse outcomes among the old population and reduces their quality of life, posing new challenges to global healthcare resources and socioeconomic development. Therefore, we urgently need to address the issue of frailty among such populations to achieve the long-term goal of maintaining their ability to care for themselves.

Frailty is generally considered to be reversible (5) Early identification and intervention can help patients regain their ability to care for themselves and avoid adverse events such as disability, depression, falls, dementia, and even death (6–8). Currently, non-pharmacological interventions are the primary approach for treating and managing frailty (9), including multi-component interventions and nutritional interventions (10, 11). However, most studies have focused solely on quantitative outcomes related to frailty. The research methods used in randomized controlled trials (RCTs) fail to accurately reflect the real-life experiences and care needs of frail older patients, overlooking the varying degrees of harm caused by the disease and the actual care needs of older patients at all stages of the frailty process. Increased frailty often portends social isolation and loneliness for older patients in the future (12). At the same time, there will be an increased demand for care provided by families, community groups, nursing homes, and hospitals. Providing practical assistance in advance can help frail older patients actively address these issues and reduce their negative emotions.

Compared with meta-analyses that routinely include quantitative studies, meta-synthesis of qualitative studies may enrich our understanding of complex, multifaceted health experiences and healthcare practice environments, increase the depth of understanding of results, and improve the openness and transparency of results (13). Therefore, this study aims to conduct a comprehensive review of qualitative research on frailty in older adults using a meta-synthesis approach to assess the real experiences and care needs of frail older adults. It provides valuable references and insights for care providers, policymakers, and medical researchers in developing intervention programs.

## 2 Methods

This meta-synthesis of systematic reviews and qualitative studies was reported under the statement “Enhancing transparency in reporting the synthesis of qualitative research” (14, 15). The study protocol was registered in PROSPERO (CRD420251038626).

### 2.1 Literature search

Computerized searches were conducted in the English-language databases PubMed, Web of Science, Scopus, Embase, CINAHL, and PsycINFO, with search dates ranging from the establishment of each database to August 25, 2024. The English retrieval terms included Qualitative Research, qualitative study, Grounded theory, interview, phenomenology, content analysis, case analysis, action research, ethnography, aged, the aged, senior citizen, old people, older, elder, agedness, senium, old age, person of advanced age, geriatric, and frailty. Using PubMed as an example, the search strategy is manifested in Appendix 1.

### 2.2 Eligibility criteria

The inclusion and exclusion criteria were determined based on the PICoS principle recommended by the Joanna Briggs Institute (JBI) Library of Evidence-Based Medicine in Australia.

Inclusion criteria: (1) study population (P): old individuals diagnosed with frailty, aged ≥60 years; (2) phenomenon of interest (I): real experiences, feelings, attitudes, and care needs of frail older patients; (3) context (Co): home, community, and related care institutions; (4) study design (S): qualitative research, including phenomenology, grounded theory, ethnography, and descriptive qualitative research.

Exclusion criteria: (1) literature for which the full text cannot be obtained or data were incomplete; (2) duplicate publications; (3) non-English literature; (4) research subjects in the terminal stage of life, suffering from major injuries, or with severe cognitive impairment; (5) studies with a quality rating of Grade C. Detailed eligibility criteria are outlined in Supplementary Table S1.

### 2.3 Literature screening and data extraction

Endnote 21 was used to remove duplicates. Full-text articles that met the inclusion criteria were retrieved, screened, and retained for review. Two researchers independently screened the literature, extracted data, and cross-checked each other’s work. In case of disagreement, they discussed or negotiated with a third researcher to resolve the issue. Data extraction included the first author, publication year, country, research method, research subjects, phenomena of interest, contextual factors, and main results.

### 2.4 Quality appraisal

Two researchers independently conducted a methodological quality assessment using the criteria developed by JBI Centre for Evidence-Based Healthcare Quality Evaluation Criteria for qualitative research (16). The assessment consisted of 10 items, each of which was evaluated as “yes,” “no,” or “unclear.” Based on the evaluation results, study quality was categorized into three grades: A, B, and C, corresponding to meeting all evaluation criteria, partially meeting evaluation criteria, and not meeting evaluation criteria, respectively. In case of disagreement, they discussed or negotiated with the third researcher.

### 2.5 Data analysis and meta-synthesis

Thomas and Harden’s three-stage thematic synthesis method was employed to summarize and integrate the included literature (17), following the systematic iterative process outlined in the JBI meta-aggregation guidelines (13). NVivo 14 software was used to assist in data extraction and narrative analysis, mainly including the following steps: (1) coding of the initial data: two researchers independently performed line-by-line coding of all included studies; (2) grouping into sub-themes: the codes were organized into descriptive themes; (3) refinement of categories: similar findings were consolidated into analytical themes; and (4) validation and consistency checking: the entire research team iteratively reviewed and discussed the resulting themes until a consensus was reached regarding interpretive sufficiency and adequacy.

### 2.6 Reflexivity

In qualitative research, the personal experiences, academic backgrounds, and methods of data collection and interpretation of the research team members are important in the research process. Therefore, transparent clarification of our position and the measures taken to manage potential biases is essential to ensure methodological rigor. PS, WXY, and KJP are clinical senior nurses with extensive experience in caring for older patients and have previously participated in qualitative research. HHZ is a graduate researcher with experience as a psychologist’s assistant. LX and TYQ are senior researchers—a nurse-in-charge and an associate chief nurse, respectively—with substantial expertise in conducting qualitative studies and systematic reviews. Together, the team brought diverse perspectives and experiences to the synthesis process and overall investigation. A critical realism view was taken when dealing with the data. Regular meetings were held to critically discuss and challenge each other’s coding decisions and emerging thematic structures. This process helped uncover and mitigate individual biases, identify key similarities/themes within the dataset (while accounting for contradictory findings), and ultimately helps us to explore this dual reality.

## 3 Results

### 3.1 Screening results

**Figure 1:**
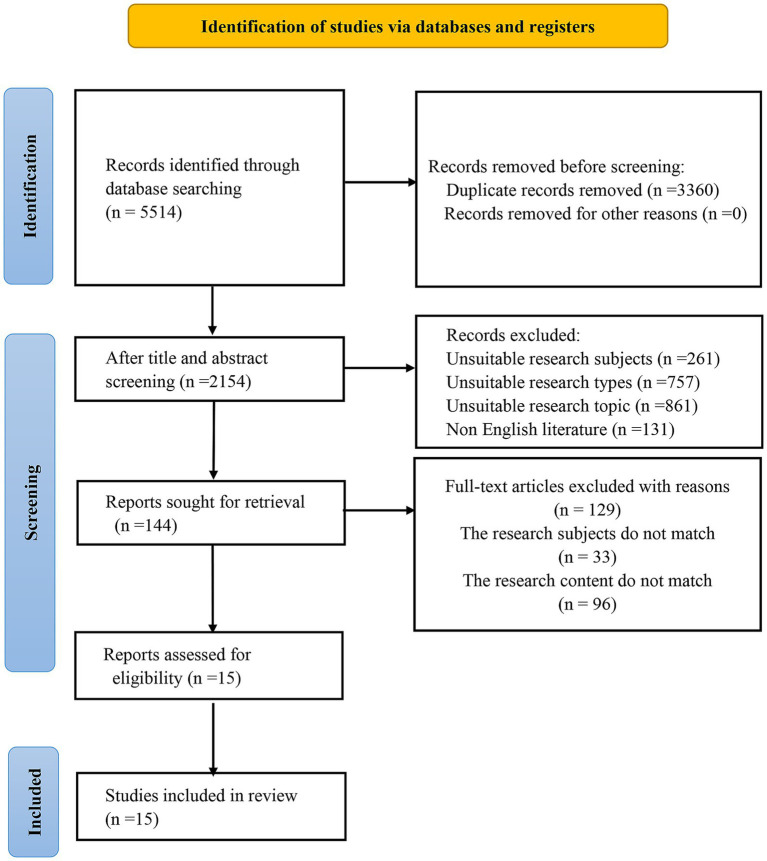
Literature screening process.

5,514 documents were retrieved, and 3,360 duplicate documents were excluded. After reading the titles and abstracts of 2,154 documents, 2,010 documents were excluded. After reading the full text of 144 documents, 129 documents were excluded because the research subjects or content did not meet the criteria. Finally, 15 documents were included. The screening process is manifested in Figure 1.

### 3.2 Basic information

15 studies involving 511 frail older patients were included. The studies were published between 2016 and 2023. Seven studies were conducted in the United Kingdom (18–24), two in the United States (25, 26), two in Switzerland (27, 28), one in New Zealand (29), one in Canada (30), one in Belgium (31), and one in Australia (32). Three studies employed descriptive research designs and methods (27, 28, 33), five used thematic analysis and semi-structured interviews (19–21, 24, 32), one combined thematic analysis with in-depth semi-structured interviews (18), one utilized a mixed-methods approach (31), one incorporated open-ended survey questions and in-depth interviews (23), one employed focus groups (26), one applied grounded theory (22), one adopted a phenomenological research approach (29), and one utilized framework analysis combined with semi-structured interviews (25). The details are listed in Table 1.

**Table 1:** Characteristics of studies included.

First author	Publication year	Country	Study design and methods	Participants	Aim	Location	Themes
Eckerblad et al.	2023	Sweden	Descriptive qualitative research	19 frail older patients with heart failure	Self-care experiences of frail older patients with heart failure	Hospital	2 themes: “To maintain my health”; “To maintain my well-being and happiness”
Warmoth et al.	2016	United Kingdom	Grounded theory	29 frail older patients	Frail older patients’ perceptions and attitudes toward frailty, including the development of frailty and its association with activity levels and health.	Participants’ homes or private meeting rooms at the first author’s university	3 themes: views on physical and psychological frailty; the process of self-identifying as frail; strategies used to resist identification.
Frost et al.	2020	United Kingdom	Thematic analysis and semi-structured interviews	28 frail older patients with anxiety or depression symptoms	Experiences of depression and anxiety among frail older patients, and perceptions of seeking help	Participants’ homes (if necessary, or at another convenient location)	6 themes: expectations of treatment; appropriateness of different treatments; promoting independence; connection; inclusivity of mental health services; endorsement of treatments.
Nair et al.	2022	United Kingdom	Thematic analysis and qualitative semi-structured interviews	28 frail older patients with different degrees of anxiety or depression	Self-management experience of depression or anxiety symptoms in frail older patients	Participants’ homes (if necessary, or at another convenient location)	4 themes: Maintaining independence; Meaning-making and recreation; Socializing and peer support; Internal mental and emotional coping strategies.
Pan et al.	2018	New Zealand	phenomenology	12 frail older patients	Frail older patients’ perceptions of frailty on physical, cognitive, and social levels	Participants’ homes or nursing homes	3 themes: the diverse conceptualizations of frailty; the neutral perception toward frailty as a concept, but the rejection of its application to themselves; the importance of independence and resilience.
Hall et al.	2019	America	Framework analysis and semi-structured interviews	12 frail older patients undergoing hemodialysis	The most important factors affecting the quality of life of frail older patients undergoing hemodialysis	Dialysis clinic or participant’s home	2 themes: having physical well-being; having social support.
Jadczak et al.	2017	Australia	Thematic analysis and semi-structured interviews	12 frail older patients or patients at risk of frailty	The views of frail older patients or those at risk of frailty on exercise recommendations, and their views on the role of general practitioners in promoting exercise among older adults.	Research centers near TQEH, TQEH Hetzel Institute, G-TRAC Center, or participants’ homes	4 themes: older people’s attitudes toward exercise; their difficulties in accessing information on exercise; the crucial role of GPs and healthcare professionals in promoting exercise; and the missing or limited advice on exercise provided by GPs.
Schoenborn et al.	2018	America	Focus groups	12 frail older patients	Frail older patients’ perceptions of frailty and information needs	A private meeting room at the medical center	3 themes: Older adults’ perception of frailty differed from the definition used in medical literature; The frail participants were more receptive to discussing frailty than non-frail or pre-frail participants. Non-frail and pre-frail participants; Informational needs about frailty
Young et al.	2022	United Kingdom	Thematic analysis and semi-structured interviews	25 frail older patients undergoing hemodialysis	Life experiences of frail older patients undergoing hemodialysis	During hemodialysis treatment or at the participants’ homes	3 themes: Factors contributing to frailty; the consequences of frailty; coping strategies; and unmet needs.
Combes et al.	2021	United Kingdom	Thematic analysis and in-depth semi-structured interviews	10 frail older patients	Barriers and facilitators to care planning for frail older patients	Participants’ homes	4 themes: Advance care planning is unclear; Lack of relevance; Importance of family, relationships, and home; Engagement strategies
Frost et al.	2018	United Kingdom	Thematic analysis and semi-structured interviews	14 mild frail older patients	Perceptions of health-promoting behaviors among mildly frail older adults	Participants’ homes	4 themes: behaviors to promote health and well-being; barriers and facilitators to health promotion behaviors; content of a health promotion service for people with mild frailty; and delivery of a health promotion service for older people with mild frailty.
Johansson et al.	2023	Sweden	Descriptive qualitative research	20 frail older patients	Daily life experiences of frail older patients and their rehabilitation experiences and perspectives	Participants’ homes or clinics	8 themes: Adaptation and strategies in everyday life; A need for help and support in activities of daily living; A decreasing social network; Experiences and perceptions of rehabilitation; The importance of staying active; Experiences of rehabilitation; Thoughts on rehabilitation; Accessibility as an obstacle to rehabilitation
Wilson et al.	2023	United Kingdom	Free-text survey questions and in-depth interviews	253 frail older patients	Experience of frail older patients with a new type of comprehensive care service	Comprehensive nursing care facility	4 themes: the overall experience of the service; interactions within the service; treatment and interventions; and outcomes due to the service.
Barnes et al.	2023	Canada	Descriptive qualitative research	15 frail older patients	Barriers and facilitators to participation in exercise rehabilitation among frail older patients	Hospital	9 themes: (1) Pre-existing conditions, fatigue, and baseline fitness; (2) Weather; (3) Guilt and frustration when unable to exercise; (4) The program being manageable and well-suited for older adults with frailty; (5) Adequate resources to support engagement with the program; (6) Support from others helps with self-perceived adherence; (7) A sense of control, intrinsic value, noticing progress and improving health outcomes; (8) Enjoyable and facilitated by previous experiences; (9) A need for individualization and variety.
Fret et al.	2019	Belgium	Quantitative questionnaire survey and qualitative semi-structured interviews	22 frail older patients in the community	Barriers to accessing formal care and support services for frail older patients in the community	Participants’ homes or local service centers	6 themes: ‘affordability’ referring to a lot of Belgian older adults having limited pensions; ‘accessibility’ going beyond geographical accessibility but also concerning waiting lists; ‘availability’ referring to the lack of having someone around; ‘adequacy’ addressing the insufficiency of motivated staff; the absence of trust in care providers influencing ‘acceptability’; and ‘awareness’ referring to limited health literacy.

### 3.3 Study quality

All studies reported consistency between philosophical foundations and methodology; consistency between methodology and research questions or objectives; consistency between methodology and data collection methods; consistency between methodology and research subjects, data analysis methods; and consistency between methodology and result interpretation methods. None of the studies explained the researchers’ circumstances from the perspective of cultural background or values. All studies described the influence of the researchers on the research and the influence of the research on the researchers. The research subjects were typical and fully reflected the research subjects and their views. Only one study was unclear as to whether it complied with current ethical standards, while the remaining 14 studies were compliant. The conclusions drawn were based on the analysis and interpretation of the data. The methodological quality of the 15 studies included was rated as Grade B. The details are listed in Table 2.

**Table 2:** Joanna Briggs Institute critical appraisal of included studies.

First author, year	1	2	3	4	5	6	7	8	9	10
Eckerblad et al. (2023) (27)	Y	Y	Y	Y	Y	N	Y	Y	Y	Y
Warmoth et al. (2016) (22)	Y	Y	Y	Y	Y	N	Y	Y	Y	Y
Frost et al. (2020) (20)	Y	Y	Y	Y	Y	N	Y	Y	Y	Y
Nair et al. (2022) (21)	Y	Y	Y	Y	Y	N	Y	Y	Y	Y
Pan et al. (2018) (29)	Y	Y	Y	Y	Y	N	Y	Y	Y	Y
Hall et al. (2019) (25)	Y	Y	Y	Y	Y	N	Y	Y	Y	Y
Jadczak et al. (2017) (32)	Y	Y	Y	Y	Y	N	Y	Y	Y	Y
Schoenborn et al. (2018) (26)	Y	Y	Y	Y	Y	N	Y	Y	Y	Y
Young et al. (2022) (24)	Y	Y	Y	Y	Y	N	Y	Y	Y	Y
Combes et al. (2021) (18)	Y	Y	Y	Y	Y	N	Y	Y	Y	Y
Frost et al. (2018) (19)	Y	Y	Y	Y	Y	N	Y	Y	Y	Y
Johansson et al. (2023) (28)	Y	Y	Y	Y	Y	N	Y	Y	Y	Y
Wilson et al. (2023) (23)	Y	Y	Y	Y	Y	N	Y	Y	Y	Y
Barnes et al. (2023) (30)	Y	Y	Y	Y	Y	N	Y	Y	Y	Y
Fret et al. (2019) (31)	Y	Y	Y	Y	Y	N	Y	Y	U	Y

Domains: 1. Congruity between the stated philosophical perspective and the research methodology. 2. Congruity between the research methodology and the research question or objectives. 3. Congruity between the research methodology and the methods used to collect data. 4. Congruity between the research methodology and the representation and analysis of data. 5. There is congruence between the research methodology and the interpretation of results. 6. Locating the researcher culturally or theoretically. 7. Influence of the researcher on the research, and vice-versa, is addressed. 8. Representation of participants and their voices. 9. Ethical approval by an appropriate body. 10. Relationship of conclusions to analysis or interpretation of the data. Code: Y: Yes N: No U: Unclear.

### 3.4 Meta-synthesis of qualitative studies

**Figure 2:**
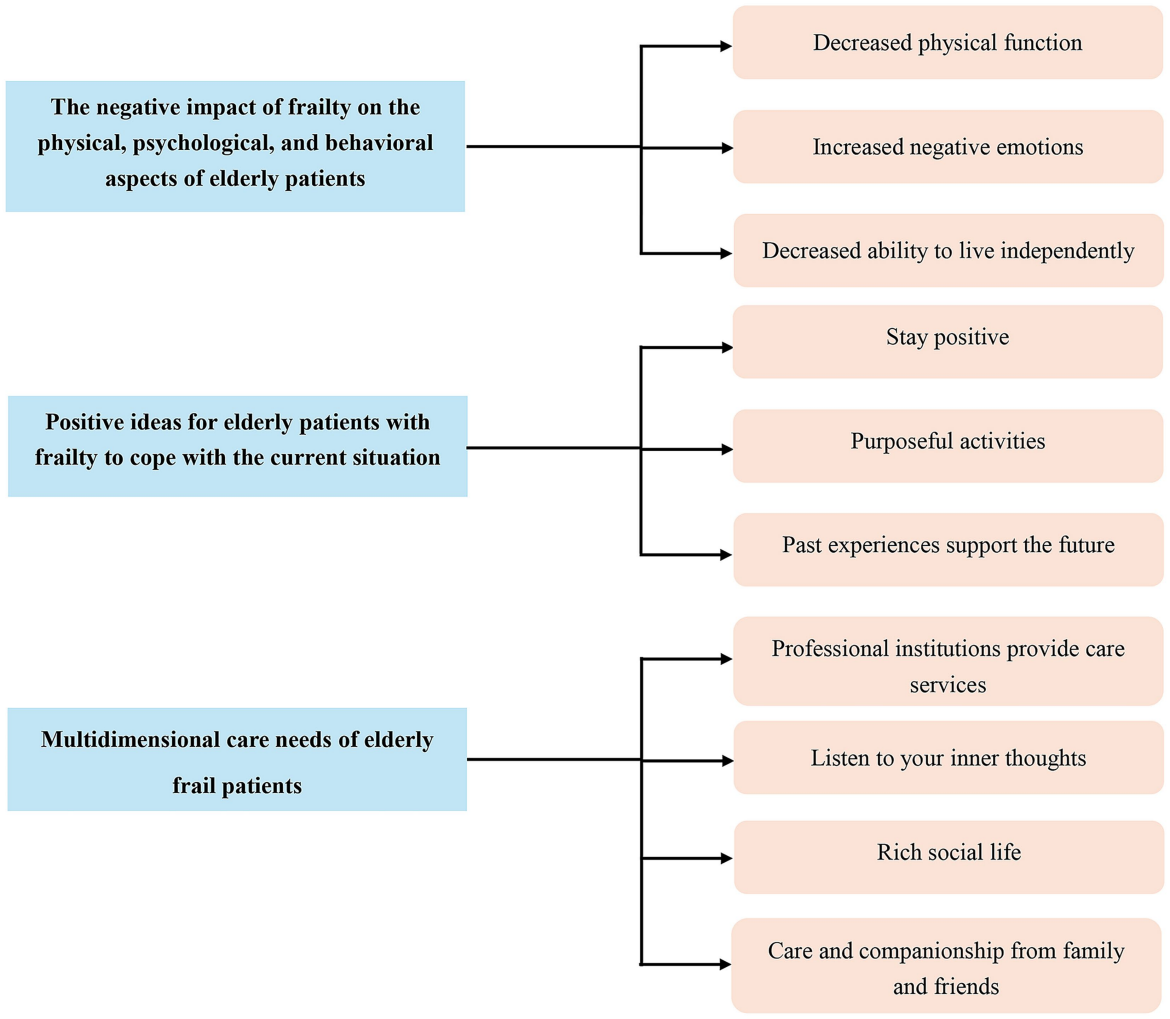
Thematic overview diagram.

Through repeated reading, analyses, and comparisons of original data, the research results were summarized into 12 categories and 3 themes. An overview of the themes is manifested in Figure 2.

#### 3.4.1 Theme 1: negative effects of frailty on the physical, psychological, and behavioral aspects of older patients

##### 3.4.1.1 Category 1: decline in physical function

Over time, frailty causes older patients to move more slowly, reduces their sensitivity to bodily sensations, and undermines the function of their sensory organs (24). Severe frailty in older adults can even manifest as recognizable clinical symptoms (22). More specific symptoms of frailty include cognitive decline, frequent fatigue, significantly increased pain, and mental exhaustion due to poor sleep quality (24, 29).

##### 3.4.1.2 Category 2: increased negative emotions

For frail older patients, the days ahead may be destined to become even more challenging (27). Some older patients even develop severe negative and pessimistic emotions (22, 24), and others’ perceptions exacerbate the stigma associated with frailty (22). They do not want to be a burden to their families (18, 24), and positive recommendations are also ignored (26). When a family member or friend suddenly passes away (29), or a doctor or other person informs them that they are experiencing frailty (26), the psychological stress caused by these events can exacerbate frailty in older patients, creating a vicious cycle. Frailty can also reduce older patients’ expectations for treatment outcomes, leading to anxiety (20).

##### 3.4.1.3 Decline in independent living abilities

The coexistence of frailty and aging makes it difficult for older patients to cope with the challenges of daily life (18). They begin to rely on tools and adopt simpler methods to deal with the problems they encounter in their daily lives (29).

#### 3.4.2 Theme 2: positive thoughts of frail older patients in coping with their current situations

##### 3.4.2.1 Category 1: maintaining a positive mindset

Frail older adults remain hopeful about their future lives, and an optimistic and positive mindset is an effective way to cope with frailty (18). In addition to their thoughts, the stimulus of people around them gives them the motivation to cope with frailty (22).

##### 3.4.2.2 Category 2: purposeful activities

Attempting to engage in and complete a new task can enhance their sense of self-identity and accomplishment. To overcome their current state of frailty, older patients need to learn to adjust and control their previous lifestyle habits and engage in more purposeful activities (19), such as exercise, paying attention to diet, social activities, improving mood and memory, and creating occupational activities (such as daily shopping).

##### 3.4.2.3 Category 3: support from past experiences

Traumatic events experienced in the past can provide frail older patients with the courage and strength to face the future. With unwavering willpower, they can confront their frail conditions (21).

#### 3.4.3 Theme 3: multidimensional care needs of frail older patients

##### 3.4.3.1 Category 1: need for professional institutions to provide care services

Frail older patients often suffer from multiple coexisting conditions, which makes them more dependent on professional medical institutions than other populations to provide medication guidance and disease management and monitoring on behalf of their families. Self-management of their health status is no longer feasible in reality (31).

In addition, to maintain basic bodily functions, frail older patients attempt to alleviate the progression of frailty through exercise, but they require official channels or clear pathways (such as videos or written records) to obtain exercise guidance (30, 32).

##### 3.4.3.2 Category 2: need for caregivers to listen to their inner thoughts

Different life experiences and social circumstances mean that they need caregivers to provide different types of psychological support, listening to their true feelings to offer emotional support (20). Effective communication can improve the quality of care and dispel their doubts about care plans (23).

##### 3.4.3.3 Category 3: need for care institutions to provide a rich social life

Frail older patients tend to lead relatively monotonous lives in their later years, so it is important to involve them in interesting social activities (19, 27). This can be achieved by setting up more spaces or venues for informal gatherings, where more people can participate and share interesting things about their lives and the challenges they face (21).

They want to participate in enriching and quiet activities such as reading newspapers, crossword puzzles, Sudoku, jigsaw puzzles, or needlework to ensure that they become better and more energetic. They want to leverage the power of the group to help them train better and restore their health (28).

##### 3.4.3.4 Category 4: need for care and companionship from family and friends

The company of family and friends is especially important for frail older patients. They are not only family members but also companions who accompany them in everything they do (19, 30). Having familiar family members or friends by their side makes patients feel warm and no longer lonely. Such moments are especially precious to them (18, 25).

## 4 Discussion

The integrated results show that frail older patients experience a decline in perception, cognition, and mobility, often accompanied by clinical symptoms such as fatigue, pain, and poor sleep quality. In addition to physical distress, they also experience corresponding negative emotions such as anxiety, psychological stress, burden, and shame, with severe cases leading to pessimistic emotions. Their ability to cope with problems in daily life declines. The above are the negative experiences of frail older patients in terms of their physical, psychological, and behavioral aspects. Of course, some optimistic frail older patients actively cope with these difficult problems in their way. They draw on their experiences of past traumatic events to support their current situation and try to maintain a positive mindset and engage in purposeful activities to slow down the process of frailty. Frail older patients need professional institutions to provide care services, caregivers to listen to their inner thoughts, care institutions to provide a rich social life, and the care and companionship of relatives and friends.

### 4.1 Dynamically assessing the physical and mental status of frail older patients and providing personalized multidimensional management plans

Frail older patients may have other diseases in addition to frailty and may be in a state of multidimensional frailty, comorbidity, or polypharmacy (34, 35). This complicates and challenges the assessment of the risks and benefits of a single chronic disease and makes it difficult to intervene with conventional care programs (36). Therefore, it is necessary to dynamically assess their physical and mental states and obtain the latest information promptly to formulate management plans tailored to their individual needs. Personalized management plans may include different levels of exercise training and equipment use, nutritional intervention plans, medication guidance, psychological intervention, and social support. An RCT demonstrated that a personalized fall prevention program based on multi-component and multi-factor interventions significantly reduced the incidence of falls among community-dwelling individuals with stroke, Parkinson’s disease, or frailty, and improved their balance function compared to standard care (37). Future studies may consider combining personalized multidimensional management programs with remote guidance and monitoring to overcome location and time constraints (38).

### 4.2 Listening to their inner thoughts and involving them in their care plans

A care management plan that is easy to adhere to and accept is even more important for frail old individuals. Involving them in the development of care plans (rehabilitation plans, dietary plans, exercise plans, daily schedules) and listening to their thoughts and feelings can help identify and reduce potential barriers to plan implementation (39). A compassionate, attentive, and polite care team can promote favorable cooperation and practices, thereby improving the quality of care (40, 41). This makes patients feel respected and understood, enabling them to receive treatment and care with dignity and follow people-oriented physical and psychological care. A qualitative study of preventive home visit services for patients over the age of 80 or with chronic illnesses showed that favorable communication skills can enhance patients’ self-esteem and make them feel that they can control their health, thereby motivating them to participate in health-promoting activities (33, 42).

### 4.3 Providing multi-channel information support

With the widespread development of information technology platforms, patients now have access to health management information in various ways. Currently, the most common method is based on the Internet of Things technology, which uses wearable devices and sensors to identify and monitor their digital biomarkers (such as gait, activity, sleep, heart rate, hand movements, and spatial mobility) to obtain more intuitive and dynamic health data (43, 44). In addition, VR technology can be combined with deep learning to enhance the immersive experience and dynamic adaptability of information platforms, thereby improving patient engagement and training compliance (45). Current research has mostly focused on getting and monitoring patient information to help prevent and control adverse events. Future research should focus on integrating these topics into broader research to meet the changing and personalized care needs of older adults.

### 4.4 Strengthening peer support and family support

Receiving care in familiar surroundings can help frail older patients feel more confident about their treatment, become more independent, and increase their motivation to achieve their rehabilitation goals. A prospective study found that “virtual contact with children less than once a week,” “loneliness,” and “social support that can provide financial assistance” were important factors affecting frailty in older adults (46). A scoping review of qualitative analysis indicated that interventions involving social engagement can enhance comfort, enjoyment, and self-worth among older adults with mild cognitive impairment (47). Therefore, it is recommended that future nursing plans consider patients’ social networks to provide maximum family and peer support. In addition, community or nursing homes can regularly hold group psychological support sessions and encourage family participation, which will help improve patients’ social weakness.

### 4.5 Strengths and limitations

To our knowledge, this is the first systematic review to propose a meta-integration of qualitative studies to comprehensively understand the real experiences and care needs of frail older patients through a multidimensional assessment. However, this study only focused on the real experiences and care needs of frail older patients and did not consider the views of caregivers or other professionals, which may affect the completeness of our findings. Future research should include more qualitative studies to explore the real experiences of frail older adults receiving care in different economic and cultural contexts. Based on these findings, targeted care measures should be developed to promote the physical and mental transition of frail older adults, alleviate their discomfort during the transition period, and improve their quality of life.

## 5 Conclusion

Our study uses meta-integration to gain an in-depth understanding of the real experiences and care needs of frail older patients. It reveals the multidimensional challenges faced by patients in physiology, psychology, and behavior. It also identifies the care needs of frail older patients in terms of professional services and social support. This suggests that care facilities and professionals should provide multi-channel, personalized, multidimensional care plans while increasing patient participation and emphasizing peer support and family support.

## Data Availability

The original contributions presented in the study are included in the article/Supplementary material, further inquiries can be directed to the corresponding author.
