# Supplementary material for: Real experiences and care needs of frail older patients: a systematic review of qualitative studies
Source: Front Public Health. 2025 Oct 1;13:1679832. doi: 10.3389/fpubh.2025.1679832 (PMC12521136; doi:10.3389/fpubh.2025.1679832)
Supplement: Supplementary file 1 [file Data_Sheet_1.zip › supplementary files/Appendix 1.docx]

**Appendix 1** Search strategy

**Pubmed**

| Search number | Search Details | Results |
| --- | --- | --- |
| 4 | (((Qualitative Research[MeSH Terms]) OR (((((((((Qualitative Research[Title/Abstract]) OR (qualitative study[Title/Abstract])) OR (Grounded theory[Title/Abstract])) OR (interview[Title/Abstract])) OR (phenomenology[Title/Abstract])) OR (Content analysis[Title/Abstract])) OR (Case analysis[Title/Abstract])) OR (action research[Title/Abstract])) OR (ethnography[Title/Abstract]))) AND ((Aged[Mesh]) OR ((((((((((((the aged[Title/Abstract]) OR (senior citizen[Title/Abstract])) OR (old people[Title/Abstract])) OR (older[Title/Abstract])) OR (elder[Title/Abstract])) OR (agedness[Title/Abstract])) OR (senium[Title/Abstract])) OR (old age[Title/Abstract])) OR (elderly people[Title/Abstract])) OR (person of advanced age[Title/Abstract])) OR (elderly[Title/Abstract])) OR (geriatric[Title/Abstract])))) AND (frailty[Title/Abstract]) | 798 |
| 3 | frailty[Title/Abstract] | 32,942 |
| 2 | (Aged[Mesh]) OR ((((((((((((the aged[Title/Abstract]) OR (senior citizen[Title/Abstract])) OR (old people[Title/Abstract])) OR (older[Title/Abstract])) OR (elder[Title/Abstract])) OR (agedness[Title/Abstract])) OR (senium[Title/Abstract])) OR (old age[Title/Abstract])) OR (elderly people[Title/Abstract])) OR (person of advanced age[Title/Abstract])) OR (elderly[Title/Abstract])) OR (geriatric[Title/Abstract])) | 4,093,217 |
| 1 | (Qualitative Research[MeSH Terms]) OR (((((((((Qualitative Research[Title/Abstract]) OR (qualitative study[Title/Abstract])) OR (Grounded theory[Title/Abstract])) OR (interview[Title/Abstract])) OR (phenomenology[Title/Abstract])) OR (Content analysis[Title/Abstract])) OR (Case analysis[Title/Abstract])) OR (action research[Title/Abstract])) OR (ethnography[Title/Abstract])) | 382,484 |
