# Supplementary material for: Real experiences and care needs of frail older patients: a systematic review of qualitative studies
Source: Front Public Health. 2025 Oct 1;13:1679832. doi: 10.3389/fpubh.2025.1679832 (PMC12521136; doi:10.3389/fpubh.2025.1679832)
Supplement: Supplementary file 1 [file Data_Sheet_1.zip › supplementary files/Table S1.docx]

| **PICOS elements** | **Inclusion criteria** | **Exclusion criteria** |
| --- | --- | --- |
| P (population) | Old individuals diagnosed with frailty, aged ≥60 years; | Research subjects in the terminal stage of life, suffering from major injuries, or with severe cognitive impairment; |
| I (phenomenon of interest) | Real experiences, feelings, attitudes, and care needs of frail older patients; | Healthcare providers' or caregivers' perspectives on care for frail older patients; |
| Co (context) | Home, community, and related care institutions; | The study is only conducted in the intensive care unit; |
| S (study design) | Qualitative research, including phenomenology, grounded theory, ethnography, and descriptive qualitative research. | (1) literature for which the full text cannot be obtained or data were incomplete; (2) duplicate publications; (3) non-English literature; (4) studies with a quality rating of Grade C. |

**Table S1 Inclusion and exclusion criteria based on PICOS elements**
